# Intracranial Aneurysm-Associated COL22A1 Variants Impair Cerebrovascular Structure and Barrier Integrity in Zebrafish

**DOI:** 10.3390/ijms27125434

**Published:** 2026-06-16

**Authors:** Vishal Y. Mardhekar, Diandra Rufin Florat, Yatharth Kakkad, Joseph P. Broderick, Saulius Sumanas

**Affiliations:** 1 Department of Pathology and Cell Biology, Morsani College of Medicine, University of South Florida, Tampa, FL 33602, USA; 2 Department of Neurology and Rehabilitation Medicine, University of Cincinnati, Cincinnati, OH 45267, USA

**Keywords:** COL22A1, intracranial aneurysm, zebrafish, blood–brain barrier, vascular stability

## Abstract

Intracranial aneurysms (IAs) represent a major clinical concern due to their risk of rupture and the resulting morbidity and mortality. Both environmental and genetic factors contribute to IA susceptibility, yet the genetic causes of IA remain poorly understood. We previously identified several single nucleotide variants (SNVs) in collagen XXII (*COL22A1*) in affected individuals with IA. However, the functional impact of these variants has not been determined, and it remains unclear whether and how they increase IA susceptibility. Here, we tested the functional effect of these variants in a zebrafish embryo model. Inducible overexpression of six human *COL22A1* SNVs increased the incidence of cranial hemorrhage in zebrafish embryos, while overexpression of wild-type *COL22A1* had no significant effect. Overexpression of DNA construct encoding COL22A1 P989L variant disrupted intracranial vascular architecture, leading to reduced vessel length, altered vascular surface parameters, and abnormal arterial patterning. Overexpression of the P989L SNV also caused pronounced vascular leakage, reduced pericyte number, and decreased expression of the tight junction proteins Claudin-5 and ZO-1. P989L SNV overexpression was also associated with increased expression of the endoplasmic reticulum stress marker *hspa5*. In silico modeling suggested that the P989L variant likely perturbs triple-helix formation in COL22A1, thereby causing protein misfolding and compromising its function. Together, these findings demonstrate the deleterious effects of IA-associated *COL22A1* variants on vascular function and stability and suggest that these variants may increase the incidence of IA in humans.

## 1. Introduction

Intracranial aneurysms (IAs) are characterized by abnormal dilation or ballooning of blood vessels within the brain and frequently result in subarachnoid hemorrhage (SAH) upon rupture. Approximately six million individuals in the United States are affected by IAs, and globally, nearly 500,000 deaths occur annually due to aneurysm rupture [1]. Ruptured aneurysms account for nearly 75% of all SAH cases [2]. The 30-day mortality rate following SAH is 30–40%, and many survivors experience long-term neurological disability [3]. Although several environmental risk factors, such as age, smoking, and alcohol, have been associated with this condition, the influence of genetic underpinnings is highly pronounced [4,5,6]. Despite this, the precise genetic mechanisms underlying IA formation and rupture remain incompletely understood. To identify genes associated with IAs, whole-exome sequencing (WES) and genome-wide association studies (GWAS) have been employed to detect single nucleotide variants (SNVs) linked to disease risk in affected populations [7,8]. These studies have identified SNVs in several genes, including EDNRA, SOX17, CDKN2BAS/ANRIL, CNNM2, KL/STARD13, and RBBP8, that are associated with an increased risk for IAs [9,10,11]. However, the prevalence of these variants in affected individuals is relatively low. Therefore, a deeper understanding of the genetic basis of IAs is essential for identifying individuals at risk and for developing preventive and therapeutic strategies to reduce the devastating consequences of intracranial hemorrhage (ICH).

**Figure 1:**
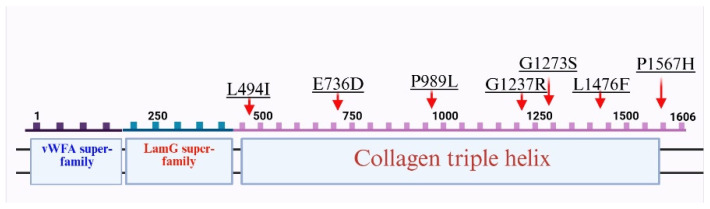
Schematic representation of the predicted domain architecture of human COL22A1, highlighting the locations of single nucleotide variants (SNVs) identified by whole-exome sequencing (WES) and targeted genome sequencing. The protein comprises an N-terminal von Willebrand factor A (vWFA) domain, a laminin G (LamG) domain, and an extended collagen triple-helix region. Red arrows indicate the positions of the identified SNVs.

Based on WES data from the familial IA studies, after filtering out common polymorphisms and prioritizing for damaging mutations predicted by PolyPhen-2 analysis [12], a small number of candidate SNVs in Collagen XXII (*COL22A1*) were identified in prior studies, including the rs142175725 single nucleotide polymorphism (SNP), which is predicted to result in an E736D substitution within the COL22A1 protein sequence [7,8,13]. COL22A1 is a member of the FACIT (fibril-associated collagens with interrupted triple helices) collagen subtype and was initially identified as a marker for the tissue or myotendinous junction (MTJ) [14]. Collagens are extracellular matrix proteins that exhibit a triple-helical structure and are pivotal constituents of the vascular wall and the basement membrane [15]. Various collagen subtypes collectively contribute to maintaining vascular stability [16]. We have previously demonstrated that *col22a1* is expressed in perivascular fibroblasts and plays a critical role in maintaining the integrity of the cranial vasculature in zebrafish [13]. Zebrafish *col22a1* mutant adults exhibited an increased incidence of ICH, which became more pronounced with age and following cardiovascular stress under forced exercise conditions, while mutant embryos showed increased incidence of hemorrhages under heat stress conditions [13]. Moreover, overexpression of wild-type human COL22A1 in zebrafish *col22a1* mutants mitigated the mutant phenotype [13]. In contrast, overexpression of the E736D variant perturbed Col22a1 function and resulted in hemorrhages in zebrafish embryos [13]. Six additional variants (predicted to result in L494I, P989L, G1273S, P1567H, G1237R, and L1476F amino acid [a.a.] substitutions) have been identified using targeted sequencing of 460 unrelated, familial IA samples via multiplex PCR, specific to the exons within the *COL22A1* locus (Figure 1) [8]. All these variants occur within the collagenous triple-helix domain of COL22A1, including residues involved in the characteristic Gly-X-Y repeats, and affect amino acids that are highly conserved across vertebrate species [13]. These variants were predicted as ‘probably damaging’ by PolyPhen-2 analysis [12], and affected individuals were heterozygous for the variants, suggesting that they may exert a dominant effect. These results suggested the possibility that mutations in *COL22A1* could be associated with an increased risk of IAs. However, it is currently not known if and how any of these additional variants perturb COL22A1 function and predispose affected individuals to IA.

The objective of this study was to investigate the functional consequences of *COL22A1* single nucleotide variants in the context of vascular stability and IA pathogenesis. To address this, we generated a zebrafish model with inducible expression of distinct human *COL22A1* SNVs and assessed their effects on cerebrovascular integrity. Our results show that expression of specific *COL22A1* SNVs increases the incidence of hemorrhage, disrupts intracranial vascular architecture, and causes pronounced vascular leakage in the brain. These phenotypes were accompanied by reduced expression of tight junction proteins and a decrease in total pericyte number. Collectively, our findings identify specific single nucleotide variants in *COL22A1*, which affect cerebrovascular stability and integrity and likely predispose affected individuals to IAs.

## 2. Results

### 2.1. COL22A1 Single Nucleotide Variants Differentially Induce Cranial Hemorrhages in Zebrafish Embryos

**Figure 2:**
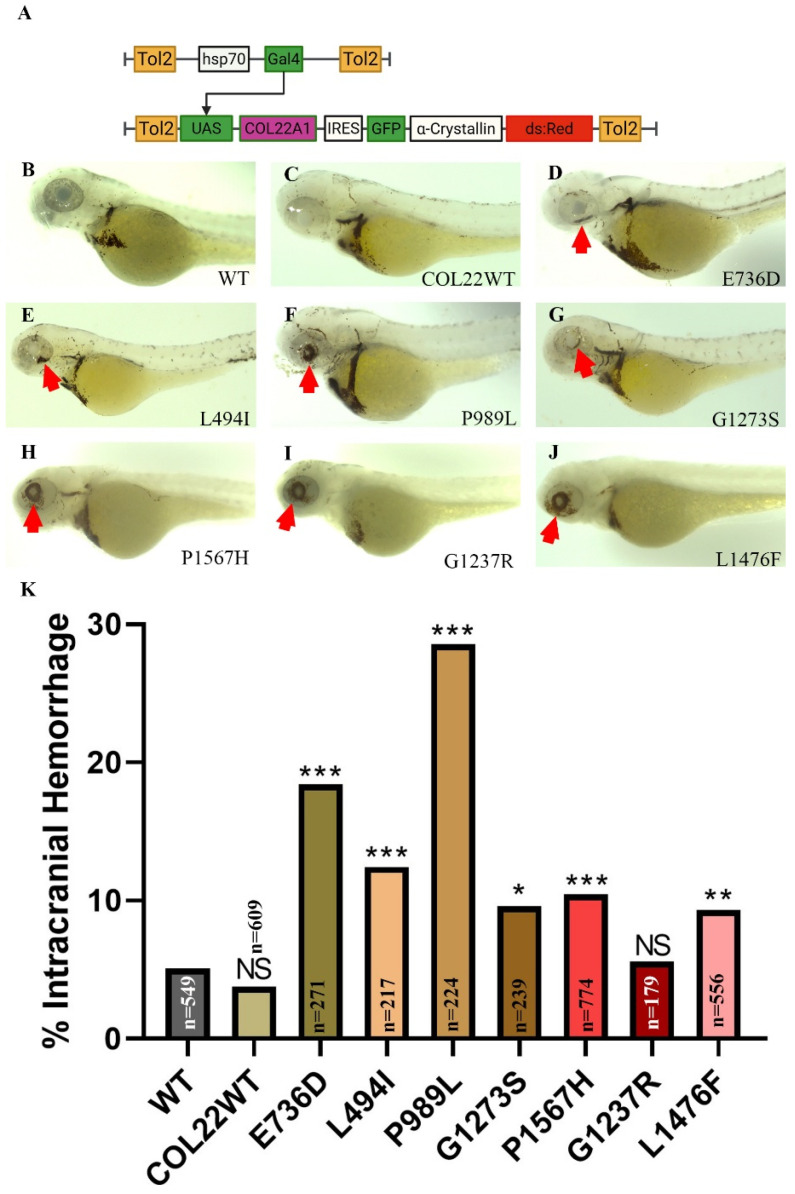
Expression of human *COL22A1* single nucleotide variants in zebrafish results in a significant increase in cranial hemorrhages. (**A**) Schematic representation of zebrafish expression of human *COL22A1* SNVs using the GAL4/UAS system. The transcriptional activator GAL4 is expressed under the heat-shock-inducible *hsp70* promoter and binds to UAS elements to drive expression of human *COL22A1* SNVs in zebrafish embryos. An α-crystallin–dsRed reporter is expressed in the lens to facilitate identification of transgenic embryos. (**B**–**J**) Representative images of WT (not transgenic), *COL22A1* WT (without hemorrhage), and embryos expressing human *COL22A1* SNVs, which correspond to different amino acid substitutions in COL22A1 protein sequence. Note the cranial hemorrhages (red arrows). Lateral view, anterior to the left, 72 hpf stage. (**K**) Quantification of cranial hemorrhage incidence at 72 hpf shows a significant increase in hemorrhage frequency in embryos expressing various human *COL22A1* SNVs compared with WT AB embryos. Statistical analysis was performed using Fisher’s exact test (* *p* < 0.05, ** *p* < 0.01, *** *p* < 0.001, NS—not significant).

As described above, seven SNVs in *COL22A1* have been previously identified in individuals with IAs (Figure 1). To determine if these SNVs contribute to cerebrovascular instability, we generated transgenic zebrafish expressing wild-type (*COL22A1* WT) and mutant human *COL22A1* SNVs under the control of upstream activating sequence (UAS) (Figure 2A). All lines were crossed to *Tg*(*hsp70:gal4*) to allow temporal control of transgene expression, using the Gal4–UAS binary system [17], in which heat-shock-induced Hsp70 promoter drives Gal4, a transcriptional activator that binds UAS to induce expression of downstream *COL22A1* transgenes. We employed a cardiovascular stress strategy as previously described [18,19]. Briefly, embryos were subjected to cardiovascular stress at 32.5 °C and heat-shocked at 24 and 48 h post-fertilization (hpf) to induce COL22A1 expression, which was confirmed by RT-qPCR (Supplementary Figure S1A,B). Cranial hemorrhages were assessed at 72 hpf using o-dianisidine staining.

Expression of six *COL22A1* SNVs produced a significant increase in hemorrhagic events compared with wild-type AB embryos (5.10%, *n* = 28/549). Among the SNVs examined, overexpression of the *COL22A1* variant encoding P989L a.a. substitution displayed the highest penetrance (28.57%, *n* = 64/224), followed by E736D (18.45%, *n* = 50/271), L494I (12.44%, *n* = 27/217), P1567H (10.47%, *n* = 81/774), G1273S (9.62%, *n* = 23/239), and L1476F (9.35%, *n* = 52/556). Hemorrhage frequency in embryos that overexpress WT *COL22A1* (3.78%, *n* = 23/609) and SNVs predicted to cause G1237R a.a. substitution (5.59%, *n* = 10/179) was not significantly different from the control wild-type AB embryos (WT) (Figure 2B–K, Supplementary Figure S1C–H). Most hemorrhages were observed around the eyes, adjacent to the lens and retina, and presumably affected ocular vasculature, while the remaining hemorrhages were present in the midbrain and hindbrain regions (Figure 2B–J, Supplementary Figure S1C–H). Despite some variability between individual clutches of embryos, mRNA expression level of most *COL22A1* SNVs was not significantly different from WT *COL22A1* expression (Supplementary Figure S1A,B), with the exception of the SNV that encodes G1237R a.a. substitution, which was expressed at a higher level than WT *COL22A1*, yet did not result in an increased incidence of hemorrhages. This argues that differences in *COL22A1* expression level cannot explain the increased incidence of hemorrhages observed in embryos overexpressing different SNVs. These data indicate that six *COL22A1* SNVs exhibit variable pathogenic potential, with the variant encoding P989L a.a. substitution being the most severe allele.

### 2.2. SNV Encoding COL22A1 P989L Disrupts Cranial Vasculature Morphogenesis

**Figure 3:**
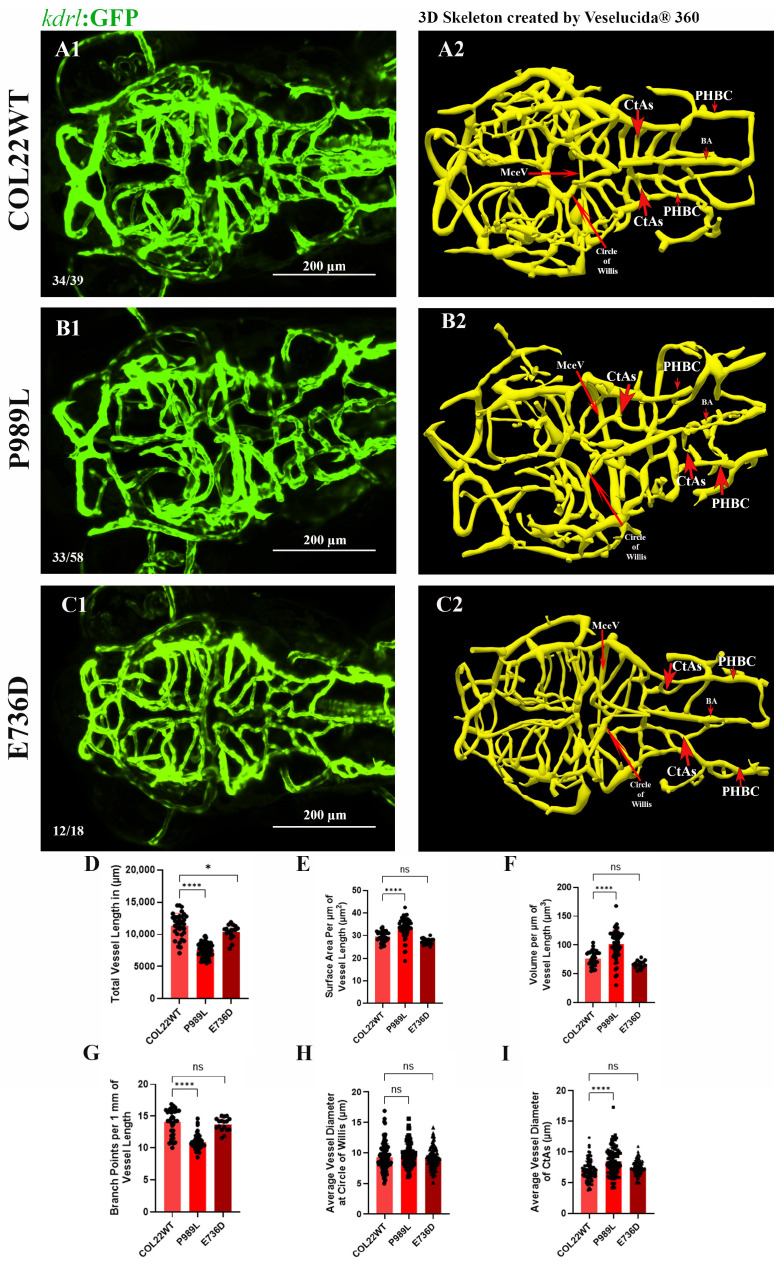
Embryos overexpressing *COL22A1* SNVs that encode P989L and E736D a.a. substitutions exhibit compromised vascular network formation. (**A1**–**C1**) Representative confocal images of intracranial vasculature in *Tg*(*kdrl:GFP*) embryos overexpressing DNA constructs corresponding to COL22A1 WT, P989L, and E736D variants, respectively, at 72 hpf. Dorsal view, anterior to the left. (**A2**–**C2**) Corresponding 3D vascular skeletons generated using Vesselucida^®^ 360. MceV—mid cerebral vein, PHBC—primordial hindbrain channel, CtAs—central arteries, BA—basilar artery. (**D**) Quantitative analysis using Vesselucida^®^ Explorer based on the 3D skeletons [*n* = 35 (*COL22A1* WT), *n* = 57 (P989L), *n* = 17 (E736D)] reveals a significant reduction in total vessel length in P989L and E736D embryos compared with COL22A1 WT. (**E**) Surface area per 1 µm of vessel length is significantly increased in the P989L group but did not change in the E736D group relative to *COL22A1* WT. (**F**) Volume per 1 µm of vessel length is significantly increased in P989L embryos and did not change in the E736D group compared with *COL22A1* WT. (**G**) The number of branch points per 1 mm of vessel length is significantly reduced in the P989L group compared with *COL22A1* WT. (**H**) No significant difference was observed in the average vessel diameter measured at the circle of Willis (100 vessels from 20 embryos for P989L, 90 vessels from 18 embryos for E736D, and 100 vessels from 20 embryos for *COL22A1* WT). (**I**) The average vessel diameter of the central arteries in the embryos predicted to overexpress the P989L variant is significantly increased when compared to the *COL22A1* WT variant (*n* = 102 vessels from 20 embryos for P989L, 94 vessels from 18 embryos for E736D, and 100 vessels from 20 embryos for *COL22A1* WT)**.** Also, note the abnormal vascular patterning of central arteries (**B1**–**C2**). The data are presented as mean ± standard deviation (SD), and statistical significance was determined using one-way ANOVA followed by Dunnett’s multiple comparisons test (* *p* < 0.05, **** *p* < 0.0001, ns—not significant).

Because overexpression of DNA constructs that encode *COL22A1* P989L and E736D a.a. substitutions resulted in the highest ICH percentage, we focused subsequent analyses on these two SNVs. To visualize blood vessels, we crossed these lines with the *Tg*(*kdrl:GFP*)*^s843^* transgenic reporter line [20], which expresses GFP in vascular endothelial cells. High-resolution confocal imaging at 3 dpf revealed pronounced vascular abnormalities in zebrafish larvae that express either of these SNVs compared with *COL22A1* WT, including a deformed vascular pattern and frequent loss of specific vascular segments, particularly central arteries (Figure 3A1–C2). Three-dimensional reconstruction analysis demonstrated a significant reduction in total vessel length in P989L (*n* = 57) and E736D (*n* = 17) SNV-expressing embryos relative to *COL22A1* WT (*n* = 35) (Figure 3D), indicating decreased vascular network density. Surface area and volume normalized per 1 µm of vessel length were significantly increased in the P989L group and did not change in the E736D group (Figure 3E,F), suggesting vessel dilation in P989L embryos. Additionally, the number of branch points per 1 mm of vessel length was significantly reduced in P989L embryos and remained unchanged in E736D embryos compared with *COL22A1* WT (Figure 3G). While vessel diameter at the circle of Willis remained comparable among groups (Figure 3H), central arteries displayed a significant increase in diameter in embryos expressing the DNA construct for the P989L variant and did not change in the E736D variant relative to *COL22A1* WT (Figure 3I).

Notably, a subset of embryos displayed atypical central artery morphology, such as excessive curvatures, discontinuous or uneven vessel extension, with irregular vascular patterning, including irregular spacing or branching of central arteries, and the absence of some central arteries. Although this phenotype was occasionally observed in *COL22A1* WT (5/39) embryos, it occurred more frequently in P989L (25/58) and E736D (6/18 embryos) (Supplementary Figure S2). Together, these findings indicate that overexpression of *COL22A1* DNA constructs that encode either P989L or E736D variants reduces cerebrovascular network density. At the same time, P989L uniquely promotes vessel enlargement and impaired branching, highlighting distinct structural consequences of these *COL22A1* SNVs on cerebrovascular morphogenesis in vivo.

### 2.3. Overexpression of P989L SNV Increases Vascular Permeability and Disrupts Tight Junction Integrity

**Figure 4:**
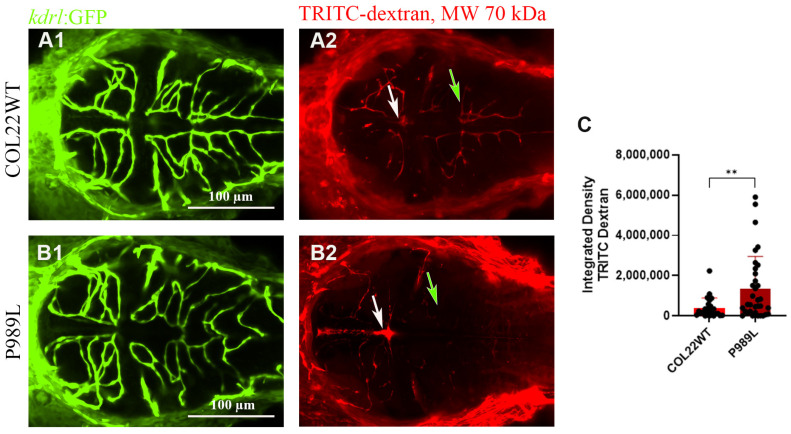
Overexpression of the *COL22A1* SNV, which encodes P989L a.a. substitution, increases vascular permeability. (**A1**–**B2**) Representative images of *COL22A1* WT (**A1**,**A2**) and P989L (**B1**,**B2**) embryos at 72 hpf expressing *kdrl:GFP*, which have been injected with TRITC-dextran (red) into the circulatory system. Dorsal view, anterior to the left. Compared with *COL22A1* WT embryos, P989L embryos show increased accumulation of TRITC-dextran in the brain ventricle (white arrows) and reduced signal within the vasculature (green arrows). (**C**) Quantification of TRITC-dextran integrated density using Fiji/ImageJ V1.54 P reveals significantly higher leakage in the ventricle area of P989L embryos relative to *COL22A1* WT controls. The data are presented as mean ± SD, and statistical significance was determined using Student’s *t*-test (** *p* < 0.01). *n* = 25 (*COL22A1* WT) and *n* = 34 (P989L).

**Figure 5:**
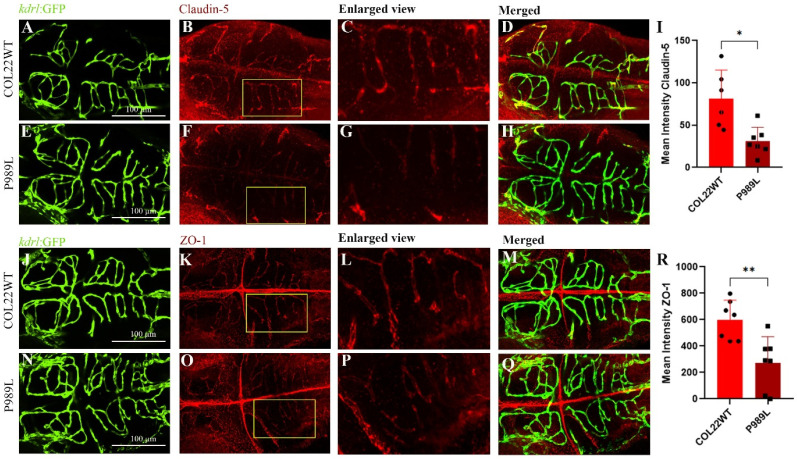
Tight junction proteins Claudin-5 and ZO-1 are significantly reduced in the *COL22A1* P989L embryos. (**A**–**D**) Representative images from the *COL22A1* WT group showing: (**A**) cranial vasculature visualized in green using the *kdrl:GFP* reporter line; (**B**) immunofluorescence staining for the tight junction protein Claudin-5; (**C**) higher-magnification view of the region outlined by the yellow box in (**B**); (**D**) merged image showing colocalization of Claudin-5 with blood vessels. (**E**–**H**) Representative images from the *COL22A1* P989L group showing: (**E**) *kdrl:GFP*-labeled vasculature; (**F**) Claudin-5 immunofluorescence; (**G**) higher-magnification view of the region outlined by the yellow box in (**F**); (**H**) merged image illustrating reduced colocalization of Claudin-5 with the vasculature. (**I**) Quantification of Claudin-5 mean gray value (MGV) demonstrates a significant reduction in Claudin-5 expression in the *COL22A1* P989L group compared with *COL22A1* WT controls (WT *n* = 6; P989L *n* = 7; Student’s *t*-test, * *p* < 0.05). (**J**–**M**) Representative images from the *COL22A1* WT group showing: (**J**) *kdrl:GFP*-labeled vasculature; (**K**) immunofluorescence staining for the tight junction protein ZO-1; (**L**) higher-magnification view of the region outlined by the yellow box in (**K**); (**M**) merged image showing colocalization of ZO-1 with blood vessels. (**N**–**Q**) Representative images from the *COL22A1* P989L group showing: (**N**) *kdrl*:GFP-labeled vasculature; (**O**) ZO-1 immunofluorescence; (**P**) higher-magnification view of the region outlined by the yellow box in (**O**); (**Q**) merged image illustrating reduced colocalization of ZO-1 with the vasculature. (**R**) Quantification of ZO-1 MGV demonstrates a significant reduction in ZO-1 expression in the *COL22A1* P989L group compared with *COL22A1* WT controls (WT *n* = 7; P989L *n* = 7; Student’s *t*-test, ** *p* < 0.01). The data are presented as mean ± SD, and all images show dorsal views of the cranial vasculature; anterior is to the left at 72 hpf.

To assess blood–brain barrier (BBB) function, *COL22A1* WT and P989L embryos were injected intravascularly with lysine-fixable tetramethylrhodamine-conjugated dextran (TRITC-dextran) at 72 hpf. Quantification of the extravascular signal revealed a significant increase in dextran leakage in P989L embryos (*n* = 34) compared with wild-type controls (*n* = 25), consistent with increased vascular permeability (Figure 4). Given this functional deficit, we examined endothelial tight junction integrity. Immunofluorescence analysis at 72 hpf demonstrated a significant reduction in signal intensity of tight junction proteins ZO-1 and Claudin-5 in cranial blood vasculature of P989L embryos (*n* = 7 for both markers) compared to *COL22A1* WT embryos (*n* = 7 and 6, respectively) (Figure 5). These data suggest that *COL22A1* SNV, which corresponds to P989L a.a. substitution, compromises BBB integrity, at least in part, by destabilizing endothelial tight junctions.

### 2.4. Overexpression of P989L SNV Results in Reduced pdgfrb mRNA Expression and Diminished Pericyte Number

**Figure 6:**
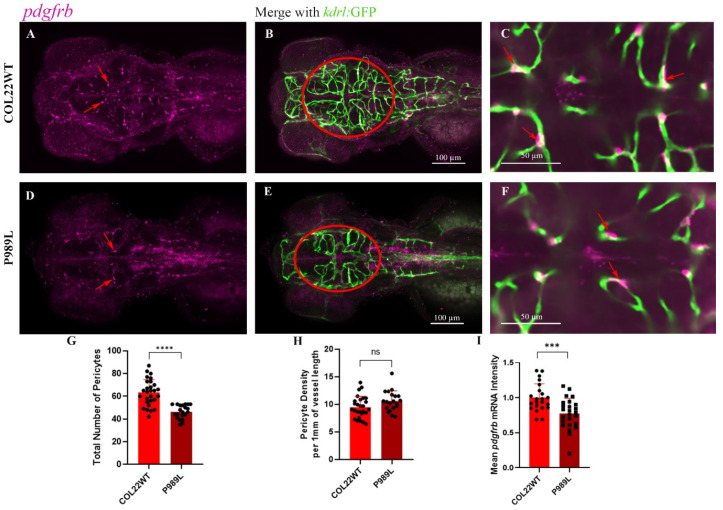
Fluorescent in situ hybridization by HCR reveals a reduction in pericyte number and *pdgfrb* mRNA intensity in the embryos overexpressing *COL22A1* SNV, which encodes P989L a.a. substitution. (**A**,**B**) Representative confocal images from the *COL22A1* WT group showing *pdgfrb* mRNA (red arrows) detected by HCR and its colocalization with blood vessels in *kdrl:GFP* reporter line. (**C**) Enlarged view of a single confocal slice from an embryo in (**B**) showing pericytes adjacent to blood vessels. (**D**,**E**) Corresponding images from the *COL22A1* P989L group at 72 hpf. (**F**) Enlarged view of a single confocal slice from an embryo in (**E**) showing pericytes adjacent to blood vessels. Dorsal view, anterior to the left in all images. Red circle in (**B**,**F**) indicates the area used for quantification). (**G**) Quantification demonstrates a significant decrease in total pericyte number in the P989L group (*n* = 21) compared with *COL22A1* WT controls (28). (**H**) When normalized to vessel length (per 1 mm of vessel), no significant difference in pericyte density is observed between the two groups. (*COL22A1* WT *n* = 27; P989L *n* = 20). (**I**) The mean intensity quantification shows a significant decrease in *pdgfrb* mRNA intensity in the P989L group (*n* = 25) compared to the *COL22A1* WT group (*n* = 22). The data are presented as mean ± SD, and statistical significance was determined using Student’s *t*-test (*** *p* < 0.001, **** *p* < 0.0001, ns—not significant).

Pericytes are crucial for blood–brain barrier integrity and vessel stability, and their loss is frequently associated with hemorrhage [21,22]. To assess whether pericyte coverage was altered in P989L embryos, we performed hybridization chain reaction (HCR) labelling of *pdgfrb*-positive mural cells at 72 hpf. The absolute number of pericytes was significantly reduced in the predefined brain area of P989L embryos, which included central arteries, compared to *COL22A1* WT embryos (Figure 6A–G), suggesting that pericyte loss could be at least partly responsible for the defects in vascular permeability and increased incidence of hemorrhages. Because P989L embryos also showed reduced cranial vasculature, we then calculated pericyte density per 1 mm of vessel length. There was no significant difference in pericyte density between the two groups (Figure 6H), suggesting that reduced pericyte number could be at least in part due to a defective vascular network in P989L embryos. Intriguingly, the mean *pdgfrb* HCR signal intensity was also significantly decreased in P989L embryos (Figure 6I), suggesting reduced pericyte *pdgfrb* mRNA expression that may reflect impaired pericyte differentiation and could contribute to the increased vascular permeability observed in these embryos.

### 2.5. In Silico Modeling Predicts Reduced Triple-Helix Stability of COL22A1 P989L

**Figure 7:**
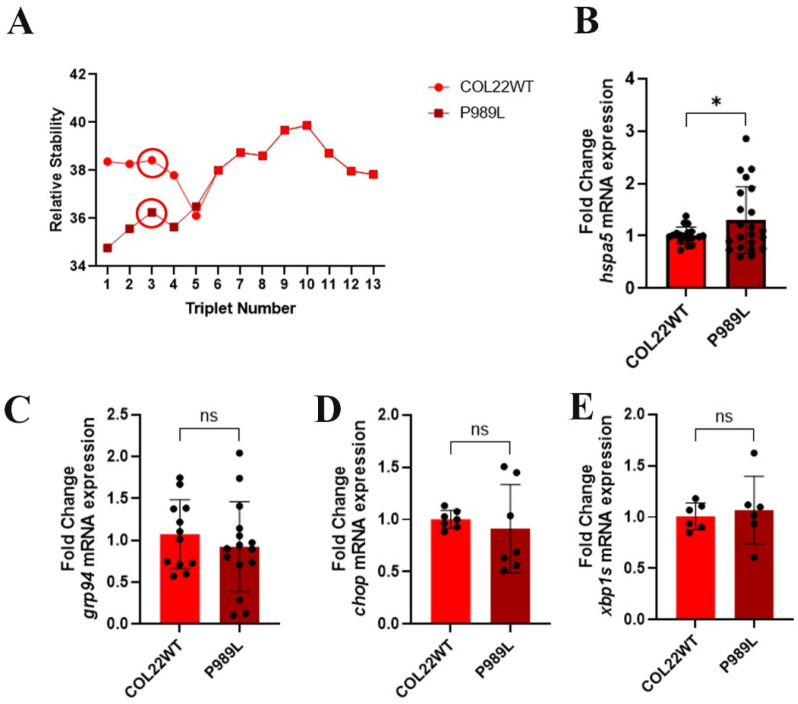
Triple-helix stability and the endoplasmic reticulum (ER) stress analysis in the P989L embryos. (**A**) Stability profile for the COL22A1 WT and the P989L generated using the online collagen stability predictor as described in the methods. The P989L variant corresponds to the second triplet of the Gly-X-Y repeat, and its relative stability is highlighted in a red circle. (**B**–**E**) RT-qPCR analysis showing fold change in expression of ER stress-related genes (*hspa5*, *grp94*, *chop*, and *xbp1s*). Among the four markers analyzed, only *hspa5* exhibits a significant increase in expression in the *COL22A1* P989L group compared with *COL22A1* WT controls, while the remaining markers show no significant change. The data are presented as mean ± SD, Student’s *t*-test, * *p* < 0.05, ns—not significant).

Collagens assemble into characteristic triple-helical structures formed by repetitive Gly-X-Y motifs, in which glycine occupies every third position. In contrast, the X and Y positions indicate proline and hydroxyproline residues that stabilize helix formation. The P989 residue is located at the Y position within this conserved Gly-X-Y repeat of the COL22A1 collagenous domain, suggesting that it contributes to maintaining proper triple-helix structure. Because this residue is evolutionarily conserved and forms part of the repetitive collagen motif, we hypothesized that the P989L substitution may destabilize the triple helix. To test the potential structural impact of this variant, we applied an established collagen stability prediction algorithm [23]. The P989L variant, which substitutes a conserved proline within this repetitive sequence, exhibited a downward shift in predicted triple-helical stability. These results suggest that the P989L substitution disrupts collagen triple-helix structure and may promote protein misfolding, potentially triggering activation of the unfolded protein response (UPR) (Figure 7A).

### 2.6. Overexpression of SNV Which Encodes P989L Partly Induces Endoplasmic Reticulum (ER) Stress

It has been previously demonstrated that misfolded collagen chains can bind to ER chaperones and activate the UPR, thereby inducing ER stress that disrupts cell function and can lead to cell death [24]. To evaluate whether expression of *COL22A1* SNV, which encodes P989L a.a. substitution, triggers ER stress, we analyzed the expression of ER stress marker genes by RT-qPCR. Analysis of WT control and P989L embryos at 72 hpf revealed a ~1.3-fold increase in *hspa5* expression (WT *n* = 18; P989L *n* = 22), whereas *grp94* (WT *n* = 10; P989L *n* = 15), *chop* (*n* = 6 per group), and *xbp1s* (*n* = 6 per group) were unchanged (Figure 7B–E). These findings suggest the limited and partial activation of UPR.

## 3. Discussion

In this study, we provide functional evidence that specific *COL22A1* single nucleotide variants identified in individuals with familial IA impair cerebrovascular stability. Using a zebrafish model with inducible overexpression, we demonstrate that several *COL22A1* SNVs act as dominant pathogenic alleles, resulting in ICH, with the variant encoding P989L a.a. substitution exhibiting the most severe cerebrovascular phenotypes upon heat-induced cardiovascular stress. Although hemorrhagic phenotypes were primarily observed in the eye/retina region, the zebrafish retina is a well-established and experimentally accessible extension of the central nervous system vasculature [25,26]. The blood–retinal barrier shares key structural and functional features with the blood–brain barrier (BBB), including tight junction architecture and conserved regulatory pathways governing vascular permeability. Prior studies have demonstrated that retinal vascular integrity in zebrafish serves as a sensitive readout of cerebrovascular stability and barrier dysfunction [13,27]. Intriguingly, zebrafish *col22a1* expression is enriched in perivascular cells surrounding the retina at 3 dpf, and also observed adjacent to the lens and retina at 1–2 dpf [13]. It is possible that *COL22A1* SNV overexpression interfered with zebrafish Col22a1 function, resulting in the reduced stability of the ocular vasculature and leading to hemorrhages.

The expression of SNVs encoding P989L a.a. substitution caused significant perturbations in vascular morphogenesis, characterized by reduced vessel length and branching, indicating defective angiogenic remodeling or impaired vessel maintenance. The observation of dilated vessels (increased surface area/volume) in P989L embryos mimics the “ballooning” morphology seen in human aneurysms, supporting the validity of this model for studying IA pathogenesis [1]. Conversely, the E736D embryos showed a less severe phenotype affecting only vessel length. This variability underscores the importance of functional validation for variants of uncertain significance identified in GWAS or sequencing studies.

Furthermore, we identified that this vascular instability is driven by disruption of the BBB, as evidenced by increased vascular permeability and loss of endothelial tight junction proteins ZO-1 and Claudin-5. This suggests impaired junctional assembly or stability, both of which are essential for restricting paracellular solute flux [28,29]. BBB breakdown is a hallmark of cerebrovascular fragility and is closely associated with aneurysm development and rupture in mammals [30]. The concordance among vascular leakage, tight junction loss, and hemorrhage strongly supports a model in which *COL22A1* SNVs compromise vessel stability primarily by perturbing endothelial–ECM interactions required for junctional maintenance.

While our initial observation suggested a decrease in pericyte numbers, normalization to total vessel length indicated that pericyte coverage remains largely proportional to the vasculature at 72 hpf. This suggests that *COL22A1* SNVs may primarily affect vascular stability rather than directly impairing pericyte recruitment. However, we observed a reduction in *pdgfrb* expression, which could indicate altered pericyte differentiation. For example, pericytes may fail to extend processes efficiently or may not cover the same vascular area, potentially leaving increased gaps along the vessel wall that could contribute to vascular permeability and instability. Notably, pericyte coverage was assessed only at 72 hpf, and potential effects on pericyte maintenance or maturation at later developmental stages were not examined.

The mechanism by which *COL22A1* SNVs cause disease appears complex. The P989L substitution introduces a bulky leucine residue into the Gly-X-Y repeat region of the collagenous domain, which our in silico modeling predicts would destabilize the triple helix. Such substitutions within the Gly-X-Y repeat are known to disrupt collagen triple-helix stability and promote protein misfolding, as demonstrated by sequence-based stability predictions and experimental studies of collagenopathies [23,31]. Consistent with this, misfolded collagen variants are frequently retained in the ER, triggering the UPR [24]. Indeed, we observed specific upregulation of *hspa5* (BiP), a master regulator of ER stress. However, other canonical UPR genes (*grp94*, *chop*, *xbp1s*) remained unchanged. Collectively, these findings suggest that ER stress is a secondary or modest consequence of *COL22A1* SNV expression rather than a primary driver of vascular pathology. An alternative possibility is that the mutant COL22A1 protein is secreted and incorporated into extracellular collagen assemblies, where it perturbs matrix structure. Collagen triple helices are composed of three α-chains, and incorporation of a mutant chain into a heterotrimer with wild-type chains could disrupt proper helix formation or stability. In this scenario, the P989L variant could act in a dominant-negative manner, compromising the integrity of the entire collagen molecule and weakening the organization of the extracellular matrix surrounding blood vessels. Such structural defects could impair the ability of the vascular matrix to withstand hemodynamic stress, ultimately predisposing vessels to instability and hemorrhage. This mechanism contrasts with other collagenopathies, such as osteogenesis imperfecta caused by type I collagen mutations or certain COL4A1-associated disorders, where ER stress and intracellular collagen retention represent major pathogenic mechanisms [32,33].

Our study has limitations. First, the use of overexpression rather than tissue-specific knock-in modeling may exaggerate dominant effects; however, the ability of wild-type human COL22A1 to rescue zebrafish *col22a1* mutants in prior studies supports the physiological relevance of this system [13]. Moreover, we confirmed expression of the introduced variants at the transcript level by RT-qPCR, demonstrating that the transgenic constructs encoding different *COL22A1* SNVs are actively transcribed in vivo. However, some variation in expression level between different batches of embryos, and different SNVs, was observed. This is likely due to the integration of *COL22A1* expression constructs in different copy numbers at different genomic sites, which is common for Tol2-mediated transgenesis [34,35]. Unfortunately, we were not able to confirm protein expression or stability due to poor quality of commercially available antibodies. Therefore, conclusions regarding variant-specific effects on protein structure and function are based on the predicted protein sequence and in silico modelling, and potential mRNA-level effects cannot be excluded. Second, while our study demonstrates vascular phenotypes, the specific molecular interactors of COL22A1 in cranial vessels remain unknown. Identifying these partners—potentially integrins, fibrillar collagens, or basement membrane components—will be critical in delineating the structural mechanisms underlying vascular instability. Future studies utilizing CRISPR-introduced knock-ins or patient-derived endothelial cells along with protein-level analyses and controlled expression studies would further validate these findings.

In conclusion, we generated a zebrafish model with inducible expression of human *COL22A1* SNVs to investigate their effects on cerebrovascular integrity. Expression of specific SNVs increased the incidence of cranial hemorrhage, disrupted vascular architecture, and caused pronounced vascular leakage in the brain. These vascular defects were associated with reduced expression of tight junction proteins, decreased pericyte numbers, and lower *pdgfrb* mRNA levels, collectively indicating impaired vascular stability. Our findings identify COL22A1 as an important regulator of cerebrovascular integrity and suggest that overexpression of several human *COL22A1* SNVs disrupts normal Col22a1 function in vivo. Together, these results support the idea that *COL22A1* SNVs identified in patients may contribute to the development and rupture of intracranial aneurysms by compromising the structural integrity of the perivascular matrix. Future studies will focus on defining the molecular mechanisms linking *COL22A1* variants to aneurysm-like vascular defects and identifying potential therapeutic strategies targeting vascular matrix stability.

## 4. Materials and Methods

### 4.1. Zebrafish Husbandry and Transgenic Line Generation

Zebrafish (*Danio rerio*) were maintained under standard laboratory conditions at 28.5 °C on a 14 h light/10 h dark cycle in accordance with institutional animal care guidelines. All zebrafish experiments were approved by the Institutional Animal Care and Use Committee (IACUC) at the University of South Florida with protocol number IS00012139 on 4 August 2023.

The *pDest-UAS: COL22A1-IRES-GFP-Tol2* construct was generated using the Gateway cloning system (Thermo Fisher Scientific, Waltham, MA, USA). The plasmid contains a 5× upstream activating sequence (UAS), full-length human wild-type *COL22A1* sequence, an internal ribosomal entry site (IRES), green fluorescent protein (GFP), and recognition sites for Tol2 transposase, as well as an *α-crystallin*: DsRed cassette for lens-specific fluorescence to enable selection of transgenic carriers [36]. Site-directed mutagenesis was performed (GenScript Inc., Piscataway, NJ, USA) to generate *COL22A1* SNVs encoding the following substitutions: E736D, L494I, P989L, G1273S, P1567H, G1237R, and L1476F, and verified using Sanger sequencing (Supplementary Figure S3). All constructs were injected into wild-type AB strain embryos together with Tol2 transposase mRNA at the one-cell stage, and stable *Tg*(*UAS:COL22A1-IRES-GFP*) lines were established based on DsRed expression in the lens. Although IRES GFP was included in the construct, GFP expression was not apparent, likely due to poor expression of genes downstream of IRES [37].

A single founder was identified for each *COL22A1* SNV and crossed to *Tg*(*hsp70:gal4*) fish [38], enabling heat-shock-inducible Gal4-driven *COL22A1* expression (Figure 2A). All embryos used in the study are derived from stable transgenic lines, each likely carrying multiple genomic integrations. Embryos were maintained at 28.5–29.0 °C until 24 hpf, then transferred to embryo medium containing 0.003% 1-phenyl-2-thiourea (PTU) to inhibit pigmentation. Beginning at 24 hpf, embryos were transferred to 32.5 °C to induce cardiovascular stress. Heat-shock induction of *COL22A1* was performed at 24 hpf and 48 hpf by transferring embryos into PCR tubes and incubating them for 30 min at 37 °C in a thermocycler. Control and experimental groups underwent identical heat-shock conditions. After heat shock, embryos were maintained at 32.5 °C until analysis. Induction of *COL22A1* expression was confirmed by RT-qPCR (Supplementary Figure S1A,B). Only *α-crystallin*: DsRed+ embryos were randomly selected for all subsequent unblinded analysis.

### 4.2. o-Dianisidine Staining and Hemorrhage Assessment

At 72 hpf, embryos were stained with o-dianisidine (Sigma-Aldrich, St. Louis, MO, USA) to detect hemoglobin as previously described [39]. Embryos were fixed in 4% paraformaldehyde in 1× phosphate-buffered saline (PBS) to facilitate long-term storage and morphological rigidity. Cranial hemorrhages were manually scored based on the presence of extravascular heme-positive signals. Fisher’s exact test was used to compare the number of embryos with or without hemorrhages between control WT AB embryos and each *COL22A1* SNP variant group, shown in Figure 2K. The raw values for the number of embryos with and without hemorrhages were used to assess statistical significance, and percentages are shown for graphical purposes.

### 4.3. Confocal Imaging and Image Processing

For live imaging, embryos were anesthetized in 0.016% tricaine and mounted in 0.6% low-melting-point agarose. Confocal images were acquired using Nikon Eclipse or Nikon AX systems (Nikon Instruments Inc., Melville, NY, USA) equipped with a 20× objective. Z-stack step size varied between experiments but was kept constant within each matched control–experimental cohort. Noise reduction was performed using the Nikon AI Denoise algorithm (NIS-Elements V5.30.02–Nikon Instruments Inc., Melville, NY, USA). Maximum-intensity projections were generated using Fiji/ImageJ V1.54 P—National Institutes of Health, Bethesda, MD, USA or Nikon Elements. Brightness and contrast adjustments were performed using the same parameters across all samples within an experiment to ensure consistency.

### 4.4. Analysis of Cranial Vasculature

At 72 hpf, live *Tg*(*kdrl:GFP*)+ embryos were imaged dorsally to visualize cranial vasculature. Three-dimensional vascular reconstructions were generated using Vesselucida^®^ 360 (MBF Bioscience V2024.1.1, Williston, VT, USA). Although we used the software’s automated reconstruction, we made manual adjustments where required to trace effectively. The tracing area was bound by the mid-cerebral vein (MceV) and the circle of Willis. Resulting skeleton maps were quantified using Vesselucida^®^ Explorer (MBF Bioscience V2024.1.1, Williston, VT, USA) to measure vessel length, vessel volume, surface area, and branch-point number. All processing parameters were applied uniformly within each experiment. Vessel diameter at the circle of Willis and central arteries (CtAs) was measured manually using ImageJ. Five measurements were obtained at each location per embryo, and all individual measurements were plotted and included in the statistical analysis.

### 4.5. Analysis of Vascular Permeability

To assess vascular leakage, 72 hpf embryos were injected with tetramethylrhodamine (TRITC)-conjugated dextran (70 kDa, lysine-fixable; Invitrogen D1818, Thermo Fisher Scientific, Waltham, MA, USA). A total of 2 µL of stock solution (25 mg/mL) was diluted in 20 µL nuclease-free water, and 3 nL was injected into the circulation at the sinus venosus. Embryos were fixed in 4% paraformaldehyde 1 h after injection and imaged by confocal microscopy. Vascular permeability was quantified in Fiji by measuring the integrated fluorescence density of TRITC-dextran within the cranial ventricles outside of blood vessels.

### 4.6. Immunofluorescence Staining

Embryos at 72 hpf were fixed overnight in 4% paraformaldehyde at 4 °C. After two washes in PBST (0.1% Tween 20 in PBS), embryos were permeabilized with Proteinase K 10 µg/mL in PBST for 30 min, followed by four washes in PBDT (1% Triton X100 and 1% DMSO in PBS). Samples were blocked for 2 h with Roche Blocking Reagent (catlog #11096176001, Roche, Indianapolis, IN, USA) and incubated with primary antibodies (1:200 in PBST) overnight at 4 °C. After washing and a second blocking step (30 min), embryos were incubated with secondary antibody (1:500 in PBST) overnight at 4 °C. The following antibodies were used: Claudin-5 (mouse monoclonal, clone 4C3C2, Thermo Fisher Scientific, catalog #35-2500, RRID: AB_2533481) and ZO-1 (mouse monoclonal, clone ZO1-1A12, Thermo Fisher Scientific, catalog #33-9100, RRID: AB_2533127). Secondary antibody for fluorescence detection was Alexa Fluor 594-conjugated donkey anti-mouse IgG (H + L) (Thermo Fisher Scientific, catalog #A-21203, RRID: AB_2535789).

Following confocal imaging, fluorescence intensity was quantified using ImageJ. For each embryo, Z-stacks of equal depth were generated for all experimental groups. Mean gray value (MGV) was measured by manually selecting regions of interest within vessels. To minimize background contribution, background fluorescence was measured in an adjacent non-vascular area for each embryo and subtracted from the corresponding vessel MGV before analysis.

### 4.7. Hybridization Chain Reaction (HCR)

Whole-mount fluorescent in situ hybridization was performed using the HCR v3.0 protocol as previously described [40]. Embryos were fixed at 72 hpf and processed following the manufacturer’s instructions (Molecular Instruments, Los Angeles, CA, USA). Fluorescently labeled probes targeting *pdgfrb* were purchased pre-designed from Molecular Instruments. Hairpin amplification was performed overnight at room temperature.

### 4.8. Pericyte Quantification

Pericytes were identified as *pdgfrb*-positive cells closely associated with cranial blood vessels using Nikon NIS-Elements software. Only *pdgfrb*-positive cells directly adjacent to the vessel surface were classified as pericytes. Quantification was restricted to a predefined anatomical region of the brain, as illustrated in Figure 6, and between the MceV and the circle of Willis region. Total pericyte number was normalized to vascular length to account for differences in vessel density. Vascular length measurements were obtained from three-dimensional reconstructions generated using Vesselucida^®^ 360. In addition to pericyte counts, fluorescence intensity of *pdgfrb*-positive cells was quantified using ImageJ. Measurements were performed on single optical planes in which individual cells were in clear focus. Regions of interest (ROIs) corresponding to individual *pdgfrb*-positive cells were manually delineated, background fluorescence was subtracted, and the MGV was recorded for each cell. Approximately ten cells were analyzed per sample.

### 4.9. Prediction of Collagen Triple-Helix Stability

Collagen triple-helix stability was assessed using an online collagen stability prediction tool (https://collagen.princeton.edu, accessed on 3 March 2026) [23] based on Gly-X-Y repeat analysis. Wild-type and mutant peptide sequences were entered using the one-letter amino acid code in accordance with the program’s input requirements. The wild-type sequence was derived from the collagen alpha-1(XXII) chain isoform X1 (Homo sapiens) (NCBI Reference Sequence: XP_011515185.1) (GKDGEPGLRGSPGLPGPLGTKGDRGAPGIPGSPGSRGDP). The corresponding mutant sequence (GKDGELGLRGSPGLPGPLGTKGDRGAPGIPGSPGSRGDP) was generated by introducing the indicated amino acid substitution. Both sequences were analyzed as homotrimeric collagen peptides of equal length. Sequences were verified to maintain an uninterrupted Gly-X-Y repeating pattern to ensure valid stability calculations. Stability profiles were generated independently for the wild-type and mutant sequences by the prediction tool. Resulting stability outputs were exported and subsequently combined manually to generate a single comparative stability plot illustrating differences between wild-type and mutant collagen peptides.

### 4.10. RNA Isolation and Quantitative Real-Time PCR

At 72 hpf, embryos were snap-frozen on dry ice. RNA extraction was performed using the RNAqueous-4PCR kit (Thermo Fisher Scientific, catalog #AM1914), and cDNA synthesis was performed using the SuperScript VILO kit (Invitrogen, catalog #11-754-050, Thermo Fisher Scientific). RT-qPCR reactions were prepared using SYBR Green Master Mix (Applied Biosystems catalog #A25742, Thermo Fisher Scientific) and run on an Azure Cielo real-time thermocycler. Melt-curve analysis confirmed the specificity of amplification. Gene expression levels for *hspa5*, *grp94*, *chop*, and *xbp1s* were normalized to *eef1a1l1/ef1α*, and fold-change values were calculated using the 2^−ΔΔCT^ method [41]. Each comparison used at least three independent biological replicates, with technical duplicates for each gene.

Primer sequences:

*ef1α*: 5′-TCACCCTGGGAGTGAAACAGC-3′, 5′-ACTTGCAGGCGATGTGAGCAG-3′
*hspa5*: 5′-CGAAGAAGCCAGATATCGATGA-3′, 5′-ACGGCTCTTTTCCGTTGAAG-3′
*grp94*: 5′-GGCGTTAATCTGCTATTGAG-3′, 5′-GTCTTTGGTTTGTCCTTGTC-3′
*chop*: 5′-CACAGACCCTGAATCAGAAG-3′, 5′-CCACGTGTCTTTTATCTCCC-3′
*xbp1s*: 5′-CAAAGGAGCAGGTTCAGGTAC-3′, 5′-GGAGATCAGACTCAGAGTCTG-3′
*COL22A1*: 5′-ATGGCCGGCCTCCGAGGG-3′, 5′-TCGGGGCCCACCTCGAAGG-3′

### 4.11. Sex as a Biological Variable

Zebrafish lack sex chromosomes and have undetermined sex at examined stages, so sex is not a study variable.

### 4.12. Statistical Analysis

Statistical analyses were performed using GraphPad Prism version 10.5.0 for Windows (GraphPad Software, Boston, MA, USA; www.graphpad.com).

## Data Availability

Data is included in the manuscript and Supplemental figures. Original data is available from the corresponding author on request.

## Supplementary Materials

The following supporting information can be downloaded at: https://www.mdpi.com/article/10.3390/ijms27125434/s1.
