# Supplementary material for: Intracranial Aneurysm-Associated COL22A1 Variants Impair Cerebrovascular Structure and Barrier Integrity in Zebrafish
Source: Int J Mol Sci. 2026 Jun 16;27(12):5434. doi: 10.3390/ijms27125434 (PMC13300400; doi:10.3390/ijms27125434)
Supplement: Supplementary file 1 [file ijms-27-05434-s001.zip › ijms-4228583-supplementary.pdf]

Supplementary Figure S1:

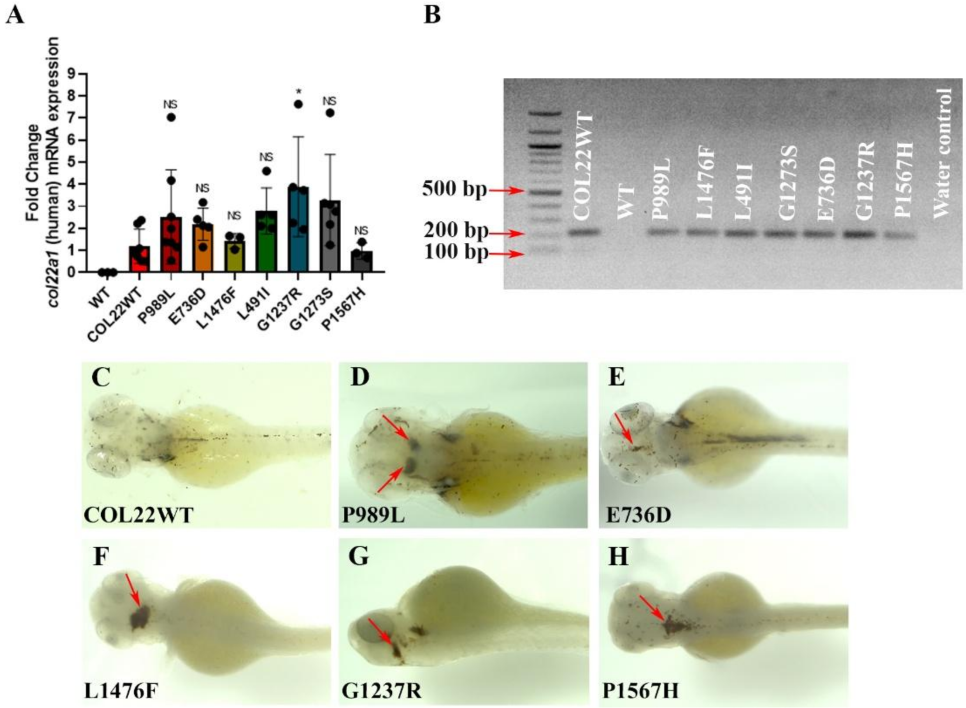

Supplementary Figure S1. Validation of overexpression of different *COL22A1* single nucleotide variants (SNVs) by RT-qPCR and additional examples of cranial hemorrhages. (A) RT-qPCR analysis showing the fold change in *COL22A1* expression. No significant differences in mRNA levels were observed between the SNVs and the COL22A1 WT overexpression group, except for G1237R SNV. Data are presented as mean ± standard deviation (SD), and statistical significance was assessed using one-way ANOVA followed by Dunnett's multiple comparisons test (*p<0.05, ns, not significant; WT n=3, COL22A1 WT n=7, P989L n=8, E736D n=5, L1476F n=3, L491I n=5, G1237R n=5, G1273S n=6, and P1567H n=3). n corresponds to the number of independent biological replicates, obtained from different batches of embryos. (B) Validation of the amplified product by agarose gel electrophoresis, showing bands at the expected size (218 bp) in the *COL22A1* WT and SNV overexpression groups, with no detectable band in the WT control. (C) *COL22A1* WT embryo without hemorrhage. (D–H) Embryos expressing SNVs that encode P989L, E736D, L1476F, G1237R, and P1567H amino acid substitutions, respectively, display cranial hemorrhages (indicated by red arrows).

Supplementary Figure S2:

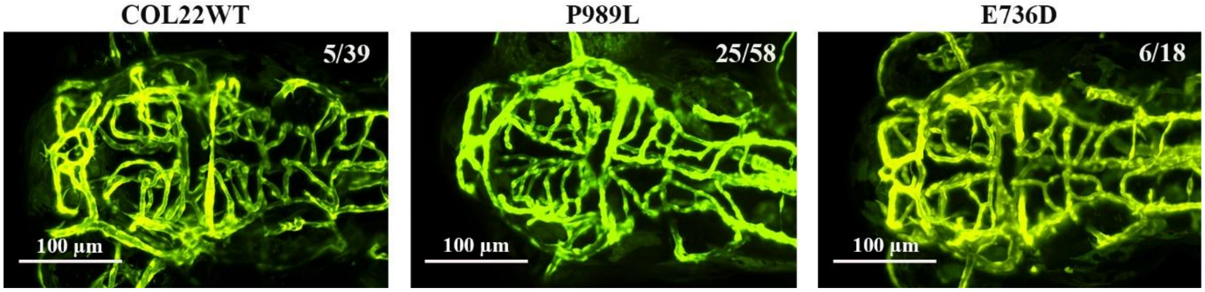

Supplementary Figure S2. Representative examples of embryos displaying atypical central artery morphology. Representative confocal images of *Tg(kdrl:GFP)* zebrafish larvae at 72 hpf showing embryos with unusual central artery morphology characterized by irregular vessel trajectories and altered vascular patterning. This phenotype was observed at low frequency in *COL22A1* WT embryos but occurred more frequently in embryos expressing the P989L SNV and in a subset of E736D embryos.

Supplementary Figure S3:

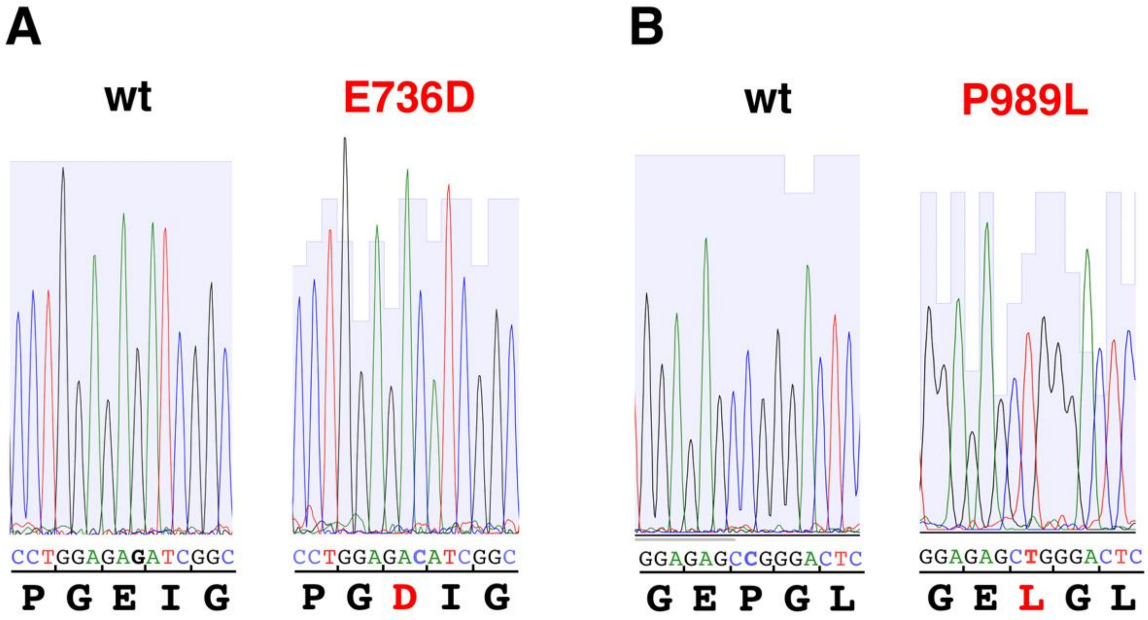

Supplementary Figure S3. Sanger sequencing verification of site-directed mutagenesis. (A) GAG to GAC substitution results in E736D substitution in the protein sequence. (B) CCG to CTG substitution results in P989L amino acid change.
